# Complementary medicine for fatigue: the importance of basic sciences and clinical studies

**DOI:** 10.2478/abm-2023-0055

**Published:** 2023-10-18

**Authors:** Editorial Office of Asian Biomedicine

**Affiliations:** Faculty of Medicine, Chulalongkorn University, Bangkok 10330, Thailand

Fatigue is a common subjective symptom, which is common in the general population and in those with illnesses. It has been generally defined as “debilitating periods of exhaustion that interfere with normal activities” [1]. The exhaustion and feelings of tiredness generally result in a decrease in physical function, mental function, or both. Both central and peripheral mechanisms can give rise to fatigue [2]. Examples of central mechanism are stress, inadequate sleep, and other psychological disturbances among others. Extensive physical activities and overwork in men and women are common mechanisms of peripheral fatigue. Fatigue can result in considerable harmful physical, psychosocial, and economic consequences that lead to poor quality of life. The subjective nature and multidimensional causes can complicate the diagnosis and the treatment of fatigue. Attempts should first be made to rule out and treat such conditions as depression, delirium, drowsiness, anemia, and muscle weakness [3]. A thorough review of medications is advisable because they can cause fatigue [4]. A complete history and a good physical examination may identify reversible causes such as anemia, ongoing blood loss, hemolysis, and folate or B12 deficiencies, as well as medication interactions. In some cases of chronic fatigue, investigations by specialists might be required to ascertain the underlying pathophysiology [5]. Once specific reversible causes are found, a specific treatment of the conditions in a particular patient can be implemented. There are several instruments to measure fatigue intensity and its functional impact [6]. Suitable measurement aiming to discern specific indicator conditions may be warranted [6] to monitor the progress of the condition and the effectiveness of treatment.

If specific factors associated with fatigue cannot be identified, some general symptomatic management such as promotion of good sleep hygiene may be tried [7]. For some conditions, such as cancer related fatigue, complementary medicine and nonpharmacologic approaches may be justified. These may include ginseng, exercise, yoga, and cognitive-behavioral therapy, as well as mindfulness-based stress reduction [8].

Hu et al.'s study [9] comprised in this volume reported the result of “screening anti-fatigue components of American ginseng saponin (AGS) by analyzing spectrum-effect relationship coupled with UPLC-Q-TOF-MS.” A total of 22 compounds from extract, and 8 prototypes constituents from serum, were identified. The constituents in serum and their anti-fatigue effects were ascertained using PLSA and gray correlation technique. The results indicated that the anti-fatigue physiological activity of AGS needed to be attributed to a mixture of compounds, and these anti-fatigue compounds include ginsenoside Re, Rb1, and Rb2. This may serve as information for future study. Other clinical and preclinical systematic reviews of ginseng derivatives also reveal compounds effective for treatment of fatigue [10].

Since natural products are widely used by patients around the world, patients and clinicians should be aware of bioactive agents that may have the potential for both benefit and harm, particularly when herb labelings of supplements are inadequate or not identified. Therefore, the value of Hu et al.'s research [9] and other similar studies lies in the fact that they offer clinicians and health care workers an important source of information that they can use in their choice of recommendation of herb-based dietary supplements to patients. Further, when patients are prescribed dietary supplements, they should always be monitored for evidence of benefits and harmful effects. As much as possible, advice about dietary supplement for fatigue should be based on the valid scientific evidence available. The evidence may be from basic sciences (such as antioxidant stress, regulating carbohydrate metabolism, delaying the accumulation of metabolites, promoting mitochondrial function, neuro-protection, anti-apoptosis, and regulating neurotransmitter disorder in central nervous system) [10]. Evidence from basic sciences will then be further strengthened through clinical studies.
